# Supplementary material for: Preparation of Sesquiterpene Lactone-Loaded PLA Nanoparticles and Evaluation of Their Antitrypanosomal Activity
Source: Molecules. 2019 Jun 4;24(11):2110. doi: 10.3390/molecules24112110 (PMC6600441; doi:10.3390/molecules24112110)
Supplement: Supplementary file 1 [file molecules-24-02110-s001.pdf]

molecules

MDPI

Article

Preparation of Sesquiterpene Lactone-Loaded PLA Nanoparticles and Evaluation of Their Antitrypanosomal Activity

Njogu M. Kimani[1], Solveig Backhaus[2], Josphat C. Matasyoh[3], Marcel Kaiser[4, 5] Fabian C. Herrmann[1], Thomas J. Schmidt[1*] and Klaus Langer[2*]

[1] Institute of Pharmaceutical Biology and Phytochemistry (IPBP), University of Muenster, PharmaCampus Corrensstrasse 48, Muenster D-48149, Germany; m_kima01@uni-muenster.de
[2] Institute of Pharmaceutical Technology and Biopharmacy, University of Muenster, PharmaCampus Corrensstrasse 48, Muenster D-48149, Germany; sbackhau@uni-muenster.de, k.langer@uni-muenster.de
[3] Department of Chemistry, Egerton University, P.O. Box 536, Egerton 20115, Kenya; josphat2001@yahoo.com
[4] Swiss Tropical and Public Health Institute (Swiss TPH), Socinstrasse 57, Basel CH-4051, Switzerland
[5] University of Basel, Petersplatz 1, Basel CH-4003, Switzerland; Email: marcel.kaiser@unibas.ch
* Correspondence: thomschm@uni-muenster.de; Tel.: +49-251-83-33378; k.langer@uni-muenster.de; Tel.: +49-251-83-39861

Received: 3 May 2019; Accepted: 31 May 2019; Published: 4 June 2019

1. Validation of HPLC methods

The HPLC methods for the determination of the amount of STL loaded into the NPs were validated in terms of specificity, linearity, precision, accuracy, stability, limit of detection (LOD), and limit of quantification (LOQ) according to International Council for Harmonisation (ICH) guideline Q2(R1) [19]. Prior to this, standard solutions were prepared as follows. The STLs stock solution of 1 mg/mL was prepared in MeCN and subsequent dilutions were carried out to obtain 9 standard solutions (10, 15, 20, 25, 30, 35, 40, 45, and 50 µg/mL). Additionally, six standard solutions (1, 2, 4, 6, 8, and 10 µg/mL) were prepared for the determination of the limit of quantification (LOQ) and the limit of detection (LOD).

HPLC analysis were performed on a JASCO (Groß-Umstadt, Germany) analytical HPLC system (pump: PU-2080 plus; autosampler: AS 2055 plus; 4-line-degasser: DG 2080-54; column oven, Jetstream plus; UV-DAD-detector: MD-2010 plus; LC Net II /ADC Chromatography Data Solutions; sample injection loop, 100 µL) on a Nucleodur C-18 ec, column (5 µm, 250 x 4.6 mm, Macherey-Nagel, Düren, Germany) with binary gradients of the mobile phase consisting of water and MeOH. ChromPass Chromatography Data System, Version 1.8.6.1 software was used in general operation, to record and analyze the chromatograms. The analysis were operated using the following specific conditions. For α-santonin, the mobile phase consisted of 40:60 H_2O-MeOH mixtures at a flow rate of 0.5 mL/min. The chromatograms were recorded at a wavelength of 244 nm. A flow rate of 0.8 mL/min and a mobile phase consisting of 20:80 H_2O-MeOH was used in the case of arglabin. A mobile phase of 50:50 H_2O-MeOH was used for the analysis of vernolepin at a flow rate of 0.7 mL/min. In the case of eucannabinolide and schkuhrin II, flow rates of 0.8 mL/min and mobile phases consisting of 40:60 and 25:75 H_2O-MeOH, respectively, were used. The chromatograms were recorded at 204 nm for arglabin, vernolepin, eucannabinolide, and schkuhrin II. Injection volumes of 10.0 µL and a column temperature of 25°C were maintained in all case.

1.1. Specificity

The specificity or selectivity was evaluated by comparing the representative chromatograms of samples containing possible interfering substances (excipients used in nanoparticle formulation) and samples containing STLs. A solution containing unloaded NP in MeCN and another containing a mixture of NPs and STLs were prepared in MeCN, centrifuged, filtered and injected into the HPLC and the chromatogram was compared with those from STLs standard solutions. Possible interferences were investigated in the chromatograms.

1.2. Linearity

The linearity was evaluated by considering a data analysis of calibration curve obtained for 9 STL standard solutions (10, 15, 20, 25, 30, 35, 40, 45, and 50 μg/mL) in MeCN. A regression analysis from the plot of the peak area versus concentration using the minimum square method was performed. The linearity was expressed as the correlation coefficient (R^2), considering values higher than 0.99.

1.3. Limit of quantification (LOQ) and detection (LOD)

The LOD and LOQ of STL solutions were determined from calibration curves prepared with 1, 2, 4, 6, 8, and 10 μg/mL STLs standard concentrations. The LOD and LOQ were based on the standard deviation (SD) of the response and the slope of the constructed calibration curve. The LOD and LOQ were calculated by the following equations:

$$LOD = 3\ \frac{\sigma}{\mathrm{s}}$$

$$LOQ = 10\ \frac{\sigma}{\mathrm{s}}$$

Where σ is the SD of the response and S is the slope of the calibration curve.

1.4. Precision

The precision was determined by the analysis of the STLs solution at three different standard solutions and over five replicates (10, 30, 50 μg/mL, n=5) on the same day (intra-day precision) and on two consecutive days (inter-day precision). The results were expressed as the standard deviation (SD) and the relative standard deviation (RSD) of the data. The acceptable values for the RSD are less than 2%

1.5. Accuracy

The accuracy was evaluated by a recovery test by calculating the percent recovery of the STLs at three concentration levels and then determining the RSD. The mean concentration value obtained for each level was compared to the theoretical value, which was considered to be 100%. The 95 to 105% are the acceptable variation in measured concentrations.

1.6. Stability

The STLs stock solutions stability were analyzed after their exposure for five days to room temperature and 4°C. They were then compared with the analysis of freshly prepared STLs stock solutions (10, 30 and 50 μg/mL). To determine post-preparative stability, samples were run immediately after preparation and after storage for 24 h in the autosampler. Each sample was analyzed in five replicates. The STLs peak areas and their retention times were compared with those of freshly prepared solutions to determine stability.

2. *In vitro* release of STLs

To determine *in vitro* release of STLs from the NPs, 1 mg NPs were incubated in 1 mL phosphate buffer (0.1 mM, pH = 7.5) at 37°C in a thermal shaker. Samples were individually prepared for each of the following points in time: 0, 0.5, 1, 3, 5, 7, 24, 36, 48 h. The samples were centrifuged at 20,000 g for 10 min and the amount of released STL in the supernatant was determined by HPLC as described above.

3. Results - HPLC method development

In order to quantify the amount of STLs incorporated into the NPs, HPLC methods were developed. These methods were then validated in terms of specificity, linearity, precision, accuracy, stability, limit of detection (LOD), and limit of quantification (LOQ). Specificity of the HPLC methods was evaluated by comparing the chromatograms of STLs standard solutions and those with potential interfering formulation constituents. To do this, blank NP were prepared and the supernatant obtained after centrifugation was diluted in MeCN, spiked with a STL and analyzed by HPLC with a method as previously described. The resulting chromatograms showed that the STLs' retention times were in agreement with those of the standard solutions. At the respective retention times of the STLs, no peaks were observed in the chromatograms of the blank NP supernatant (Figures S1-S5). This confirmed that there was no interference in the quantitative determination of the STLs from the NP formulation constituents. These methods can thus be considered highly specific because no potential interfering peaks were observed in all cases.

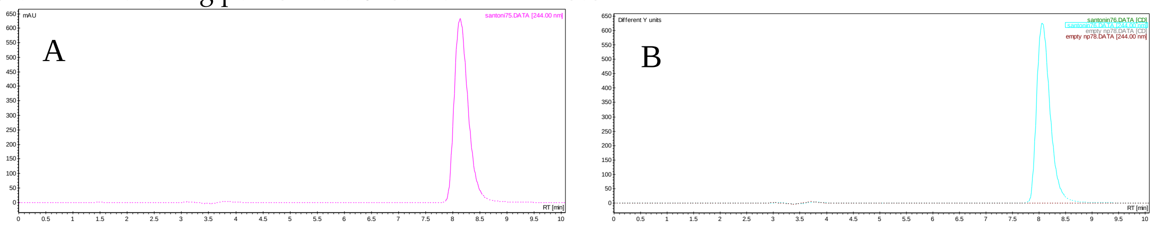

Figure S1. Representative HPLC chromatograms. A: *α*-santonin (10 μg/mL), B: *α*-santonin (10 μg/mL, green) and supernatant of empty NPs (brown).

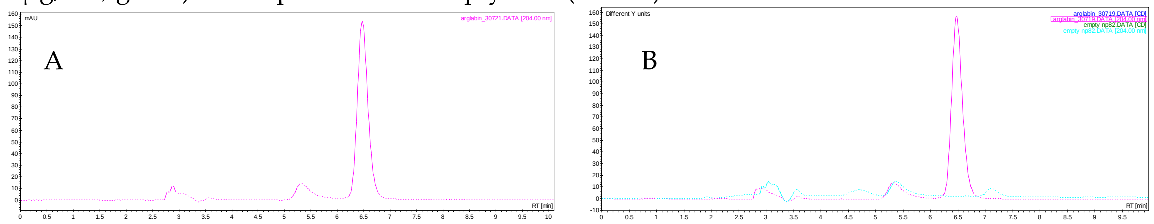

Figure S2. Representative HPLC chromatograms. A: arglabin (10 μg/mL), B: arglabin (10 μg/mL, purple) and supernatant of empty NPs (green).

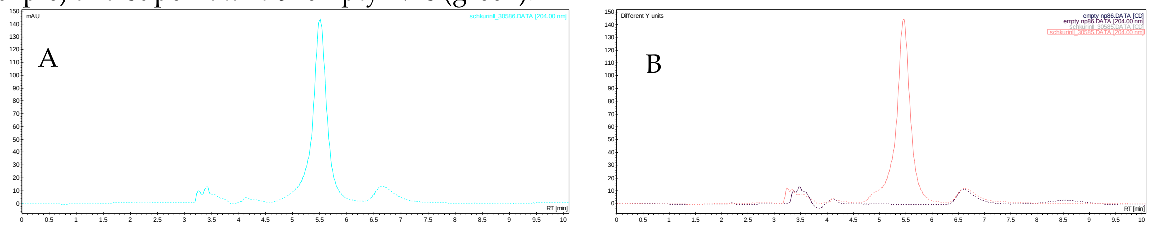

Figure S3. Representative HPLC chromatograms. A: schkuhrin II (10 μg/mL), B: schkuhrin (10 μg/mL, pink) and supernatant of empty NPs (black).

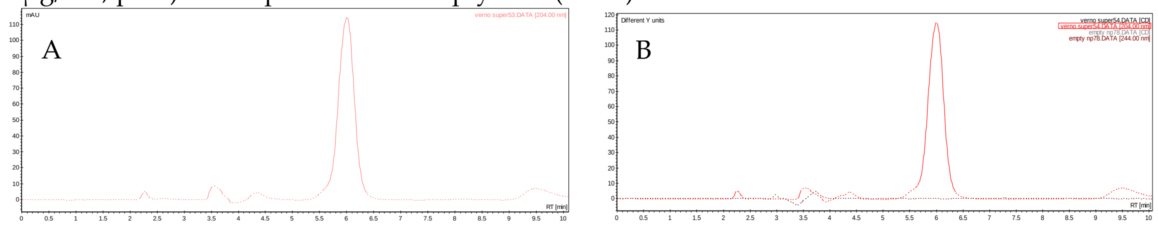

Figure S4. Representative HPLC chromatograms. A: vernolepin (10 μg/mL), B: vernolepin (10 μg/mL, red) and supernatant of empty NPs (brown).

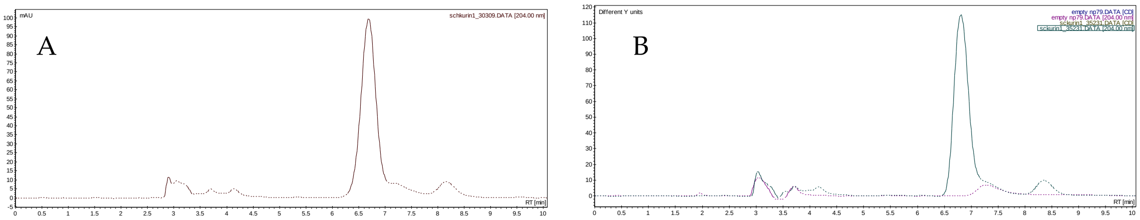

Figure S5. HPLC chromatograms. A: eucannabinolide (10 μg/mL), B: eucannabinolide (10 μg/mL, dark green) and supernatant of empty NPs (purple).

Linear relationships were found between the concentration of the STLs and the response of the HPLC quantification method in the range of 10-50 μg/mL. The regression analysis data are shown in Table S1. No significant deviation of linearity was detected at this condition. The calibration curves are shown in Figure S6.

The lowest concentration at which an analyte can be detected (LOD) or quantified (LOQ) with acceptable precision and accuracy was calculated from SD of the response and the slope obtained from linear regression of a specific calibration curve (1-10 μg/mL) in the low end of the proposed range. The methods were found to be linear. The LOD and LOQ are shown in Table S1.

Table S1.Linearity data and quantification limits of STLs

Validation parameters	α-santonin	arglabin	eucannabinolide	vernolepin	schkuhrin II
R^2	0.99	0.99	0.99	0.99	0.99
Slope ± SD (peak Area/Conc.)	2.08 ± 0.00	1.18± 0.01	1.19 ± 0.02	1.68 ± 0.02	1.67 ± 0.02
Intercept ± SD	-0.94± 0.09	1.30± 0.42	-1.43 ± 0.53	1.00 ± 0.70	-1.97± 0.63
LOD (μg/mL)	0.12	0.17	0.22	0.13	0.15
LOQ (μg/mL)	0.36	0.51	0.68	0.40	0.46

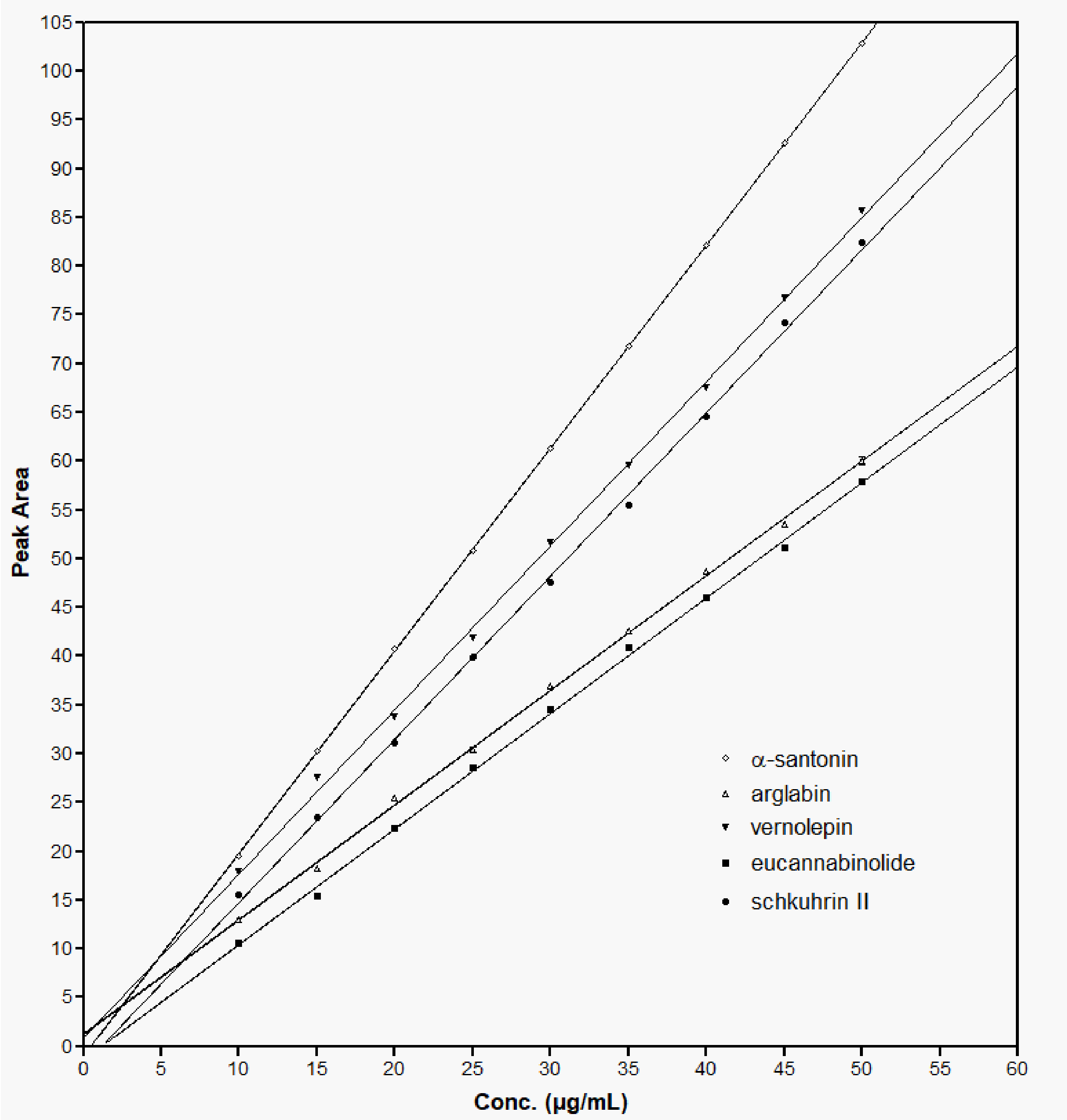

Figure S6. Calibration curves of STLs standard solutions used in the method validation

The data of precision, a measure of the relative errors of the method, expressed as the relative standard deviation (RSD) for repeatability and intermediate precision, are shown in Table S2.

Intraday and two interday precision of the methods were determined at three different days and concentrations, i.e., 10, 30 and 50 μg/mL. The RSDs of the responses were calculated for each case. All concentrations of STLs analyzed under all evaluated conditions were within the acceptable range with 1.37% being the highest RSD value obtained.

The accuracy of the methods was assessed by calculating the percent recovery and the RSD of the mean concentration of the analyte at three different concentrations (Table S2). The results display agreement between the theoretical and experimental values. This method exhibited mean recoveries (n=5) within the acceptable accuracy range (95 to 105%).

The stability of the STLs' standard solutions under experimental solutions was evaluated at three concentrations for all the STLs. This was meant to ascertain the influence of small changes to the analytical procedure on the response. The stability was evaluated based on the percent recovery and RSD values obtained using different parameters. Detailed results are shown in Table S3. This indicates that the solutions are stable within the given period (Table S3). No signs of product degradation or drug loss were observed thus confirming the stability of the STLs.

Table S2. Precision and accuracy results for the different levels of STLs in the standard solution

	**α-Santonin**			**Eucannabinolide**			**Vernolepin**			**Schkuhrin II**			**Arglabin**		
**Nominal[1]**	**Mean measured[1]**	**Precision (RSD)**	**Accuracy (%)**	**Mean measured[1]**	**Precision (RSD)**	**Accuracy (%)**	**Mean measured[1]**	**Precision (RSD)**	**Accuracy (%)**	**Mean measured[1]**	**Precision (RSD)**	**Accuracy (%)**	**Mean measured[1]**	**Precision (RSD)**	**Accuracy (%)**
**Intra-day (n=5)**															
10	9.87±0.11	1.12	98.66	9.61±0.05	0.48	96.14	9.93±0.07	0.66	99.34	10.20±0.94	0.93	101.99	10.23±0.14	1.37	102.26
30	29.96±0.08	0.26	99.87	29.78±0.16	0.54	99.28	30.2±0.21	0.71	100.66	29.96±0.37	1.24	99.86	30.07±0.31	1.03	100.24
50	50.16±0.20	0.39	100.33	50.12±0.21	0.41	100.24	50.11±0.14	0.27	100.23	50.35±0.17	0.34	100.71	50.31 ±0.47	0.94	100.62
**Inter-day 1 (n=5)**															
10	10.11±0.09	0.86	101.06	9.90±0.07	0.70	99.01	9.95±0.05	0.49	99.46	10.18±0.09	0.90	101.79	9.99 ± 0.10	0.97	99.88
30	29.74±0.11	0.37	99.14	30.1±0.12	0.40	100.18	30.32±0.09	0.31	101.05	29.88±0.29	0.97	99.61	30.23±0.16	0.54	100.75
50	51.06±0.07	0.14	102.12	49.8±0.16	0.32	99.61	50.49±0.38	0.76	100.98	50.49±0.23	0.46	100.98	50.21±0.56	1.11	100.42
**Inter-day 2 (n=5)**															
10	10.37±0.10	0.97	103.71	10.15±0.06	0.59	101.54	9.98±0.10	1.00	99.82	10.11±0.04	0.42	101.09	10.06±0.11	1.10	100.56
30	30.27±0.07	0.24	100.90	29.8±0.26	0.87	99.33	30.39±0.10	0.32	101.29	30.49±0.23	0.74	101.63	30.16 ± 0.3	0.99	100.53
50	50.82±0.14	0.28	101.64	49.33±0.21	0.43	98.66	50.45±0.09	0.187	100.89	50.19±0.13	0.25	100.39	50.55±0.57	1.14	101.10

[1](μg/mL)

Table S3. Results of the stability analysis of the STLs at different temperatures.

	α-Santonin			Eucannabinolide			Vernolepin			Schkuhrin II			Arglabin		
**Nominal[1]**	**Mean measured[1]**	**Mean stability[1]**	**RSD (%)**	**Mean measured[1]**	**Mean stabilit**	**RSD (%)**	**Mean measured[1]**	**Mean stabilit**	**RSD (%)**	**Mean measured [1]**	**Mean stability[1]**	**RSD (%)**	**Mean measured [1]**	**Mean stability[1]**	**RSD (%)**
**Stock solution stability at room temperature (n=5)**															
10	9.89±0.13	9.95±0.08	0.81	9.92±0.07	9.75±0.07	0.72	10.10±0.09	9.71±0.06	0.61	10.44±0.22	10.09±0.05	0.50	10.24±0.10	9.99±0.10	0.97
30	29.98±0.05	30.08±0.05	0.18	29.65±0.21	30.22±0.13	0.44	30.20±0.21	30.32±0.27	0.90	29.60±0.12	30.09±0.06	0.20	30.31±0.15	30.40±0.20	0.67
50	50.14±0.08	50.88±0.06	0.12	50.27±0.11	50.51±0.13	0.25	50.45±0.27	51.28±0.37	0.73	49.97±0.15	50.18±0.05	0.10	50.02±0.82	50.35±0.53	1.05
**Stock solution stability at 4°C (n=5)**															
10	9.89±0.13	10.42±0.07	0.68	9.92±0.07	10.68±0.11	1.03	10.10±0.09	10.01±0.06	0.60	10.44±0.22	9.96±0.06	0.60	10.24±0.10	9.90±0.08	0.77
30	29.98±0.05	30.5±0.16	0.53	29.65±0.21	29.50±0.14	0.48	30.20±0.21	30.49± 0.16	0.52	29.60±0.12	30.01±0.18	0.61	30.31±0.15	29.82±0.10	0.33
50	50.14 ± 0.08	49.95±0.12	0.23	50.27±0.11	49.77±0.16	0.33	50.45±0.27	51.10±0.16	0.31	49.97±0.15	49.80±0.13	0.27	50.02±0.82	50.91±0.22	0.43
**Stock solution stability in the auto sampler (n=5)**															
10	9.89±0.13	10.02±0.08	0.79	9.92±0.07	10.22±0.11	1.08	10.10±0.09	10.34±0.09	0.88	10.44±0.22	9.95±0.05	0.50	10.24±0.10	10.16±0.10	0.96
30	29.98±0.05	29.79±0.29	0.99	29.65±0.21	30.37 0.07	0.23	30.20±0.21	29.84±0.06	0.20	29.60±0.12	29.88±0.23	0.78	30.31±0.15	31.42±0.27	0.84
50	50.14±0.08	50.91±0.08	0.16	50.27±0.11	50.63±0.08	0.17	50.45±0.27	50.57±0.51	1.01	49.97±0.15	50.56±0.07	0.13	50.02±0.82	51.03±0.34	0.67

[1](μg/mL)

The physicochemical parameters log*P*, logS, p*K*a, topological polar surface area (TPSA) of the STLs and PLA were calculated from the web based ChemAxion standalone application Chemicalize available at https://chemaxon.com/products/chemicalize. These parameters are listed on Table S4 below.

Table S4. Physicochemical parameters of the STLs and PLA

	**TPSA (Å^2)**	**LogP**	**logS**	**p*K*a (strongest acidic point)**
α-santonin	43.37	2.60	-3.45	No ionizable atoms
Arglabin	38.83	2.17	-2.82	No ionizable atoms
Schkuhrin II	139.59	1.97	-3.22	12.49
Vernolepin	72.83	1.06	-1.88	14.75
Eucannabinolide	119.36	1.33	-2.38	14.79
PLA	46.53	0.17	0.22	3.99
